# XRCC7 rs#7003908 Polymorphism and *Helicobacter pylori* Infection-Related Gastric Antrum Adenocarcinoma

**DOI:** 10.1155/2013/124612

**Published:** 2013-11-11

**Authors:** Chao Wang, Xiao-Ying Huang, Jin-Guang Yao, Bing-Chen Huang, Cen-Han Huang, Pinhu Liao, Xi-Dai Long

**Affiliations:** ^1^Department of Medicine, The Affiliated Hospital of Youjiang Medical College for Nationalities (AHYMCN), Baise 533000, China; ^2^Department of Pathology, The Affiliated Hospital of Youjiang Medical College for Nationalities (AHYMCN), Baise 533000, China; ^3^Department of Liver Surgery, The Affiliated Ren Jin Hospital, Shanghai Jiao Tong University School of Medicine, Shanghai 200127, China

## Abstract

The X-ray repair cross-complementing group 7 (XRCC7) plays a key role in DNA repair that protects against genetic instability and carcinogenesis. To determine whether XRCC7 rs#7003908 polymorphism (XRCC7P) is associated with *Helicobacter pylori* (*H. pylori*) infection-related gastric antrum adenocarcinoma (GAA) risk, we conducted a hospital-based case-control study, including 642 patients with pathologically confirmed GAA and 927 individually matched controls without any evidence of tumours or precancerous lesions, among Guangxi population. Increased risks of GAA were observed for individuals with cagA positive (odds ratio (OR) 6.38; 95% confidence interval (CI) 5.03–8.09). We also found that these individuals with the genotypes of XRCC7 rs#7003908 G alleles (XRCC7-TG or -GG) featured increasing risk of GAA (ORs 2.80 and 5.13, resp.), compared with the homozygote of XRCC7 rs#7003908 T alleles (XRCC7-TT). GAA risk, moreover, did appear to differ more significantly among individuals featuring cagA-positive status, whose adjusted ORs (95% CIs) were 15.74 (10.89–22.77) for XRCC7-TG and 38.49 (22.82–64.93) for XRCC7-GG, respectively. Additionally, this polymorphism multiplicatively interacted with XRCC3 codon 241 polymorphism with respect to HCC risk (OR_interaction_ = 1.49). These results suggest that XRCC7P may be associated with the risk of Guangxiese GAA related to cagA.

## 1. Introduction

Gastric cancer is the fourth most common cancer worldwide and the second most common cause of death from cancer [1, 2]. The predominant type of this tumor in Guangxi Zhuang Autonomous Region is gastric antrum adenocarcinoma (GAA) [3]. Clinic-epidemiological evidence suggests that *Helicobacter pylori* (*H. pylori*) infection is a major risk factor [4, 5]. Increasing evidence has shown that cagA protein, an important *H. pylori*-produced virulent factor for gastric mucosa injury, could induce many kinds of DNA damage including DNA base damage, DNA double-strand break (DSBs), and oxidative damage [5–10]. Among these DNA damages, DSBs are the most detrimental form [11, 12], because they may lead to both chromosomal breakage and rearrangement and ultimately lead to tumorigenesis of cancers such as GAA.

DNA repair gene “X-ray repair cross-complementing group 7” (XRCC7) is necessary for DNA ligation in the nonhomologous end-joining (NHEJ) pathway, which is responsible for repairing most double-strand breaks [13, 14]. Recently, several studies have shown that XRCC7 rs#7003908 polymorphism (XRCC7P) may be associated with DNA repair capacity and tumor risk [15–17]. However, the association between this polymorphism and GAA has not yet been elucidated. Here, we evaluated whether XRCC7P modifies *H. pylori* infection-related GAA risk.

## 2. Materials and Methods

### 2.1. Study Population

We conducted a hospital-based case-control study that has been previously described [18, 19]. Briefly, all participants, consisting of 642 GAA patients (including 465 previously studied subjects [18]) and 927 control individuals (including 739 previously studied subjects [18]), were from Guangxi Zhuang Autonomous Region, and both case and control recruitment is still ongoing. Cases were patients diagnosed with histopathologically confirmed GAA in the Affiliated Hospitals of the three main medical colleges in Guangxi (viz. Guangxi Medical University, Youjiang Medical College for Nationalities, and Guilin Medical College) between September 2008 and December 2010. During the same period, controls without clinical evidence of precancerous lesions or tumors were randomly selected from a pool of volunteers accepting the examination of gastric biopsy because of extended gastric inflammation (superficial chronic gastritis diagnosed by histopathological examination) in the same hospitals. Controls were individually matched to cases by age (±5 years), ethnicity (Han, Minority), sex, smoking habits (never smoking, ever smoking), and alcohol intake (never drinking, ever drinking). In this study, there is one or two controls (who have the aforementioned matching criteria) individually matched to each case. After giving written consent, participants provided demographic information (including age, race, medical history for themselves and their families, and drinking and smoking history) using a standard interviewer-administered questionnaire. A total of 642 cases and 927 controls, representing 99% of eligible cases and 95% of eligible controls, were enrolled and interviewed. The protocol of the study was approved by the Ethics Committees of the hospitals involved in the study.

### 2.2. DNA Extraction

DNA was extracted from noncancerous gastric biopsy tissues from all cancer patients and control subjects for deparaffinization and proteinase K digestion, described in our previously published study [19]. DNA was stored at −20°C until additional analysis.

### 2.3. *H. pylori* Infection-Status Assay

Because our previous report showed that carriers of *H. pylori* having CagA-present strains would face higher risk of GAA, we evaluated the *H. pylori*-infection status using cagA-status analysis in this study. CagA status was elucidated through the previously published PCR technique [19]. Briefly, PCR reactions were run in a 25 *μ*L final volume containing 0.625 u of GoTaq DNA polymerase (Promega Corporation, Madison, WI), 0.2 *μ*M of each primer, 200 *μ*M of each dNTPs, and about 100 ng of genomic DNA. Cycling conditions were as follows: PCR program initiated by a 2 min denaturation step at 95°C, followed by 40 cycles of 95°C for 10 s, 54.5°C for 30 s, 72°C for 20 s, and a final elongation step of 72°C for 10 min. The quality control for cagA-status assays was administered by negative and positive controls.

### 2.4. XRCC7 Genotyping

Laboratory personnel were blinded to case and control status. DNA was extracted from noncancerous gastric biopsy tissues from all cancer patients and control subjects according to the previously described methods [17]. XRCC7 (rs#7003908) genotypes were tested by using the TaqMan-PCR on iCycler iQ real-time PCR detection system (CFX Manager Version 2, Bio-Rad). The primer and probe sequences used for rs#7003908 polymorphism have previously been reported. PCR reactions were run in a 25 *μ*L final volume containing 1 × TaqMan Universal Master Mix II (catalog # 4440041, ABI), 0.2 *μ*M of each probe, 0.2 *μ*M of each primer, and about 100 ng of genomic DNA. Cycling conditions were 95°C for 10 min and 40 cycles of 95°C for 15 s and 60°C for 1 min. To further validate the genotyping results, direct sequencing was randomly tested in about 1% of PCR products, and genotyping was repeated randomly in 10% of the samples. 

### 2.5. Statistical Analysis

All statistical analyses were done using the statistical package for social science (SPSS) version 18 (SPSS Institute, Chicago, IL). The two-sided chi-square test was used to evaluate differences in frequency distributions of demographic characteristics, cagA status, and genotypes of XRCC7 rs#7003908 polymorphism between the cases and controls. Based to individually matched design, conditional logistic regression analyses were carried out to obtain odds ratios (ORs) for risk of GAA and their 95% confidence intervals (CIs). Joint effects between genotypes and *H. pylori*-infection information were evaluated by using the full regression model that included the main effects of potential risk genotypes, cagA status, and all matching factors with a fully parameterized model (not including interactive variables of both cagA and genotypes). In the present study, the interactive effects between cagA and risk genotypes of XRCC7 rs#7003908 were evaluated according to the following formula [20–22]:

(1) $$\begin{array}{c} \text{O}{\text{R}}_{\text{eg}}<\text{O}{\text{R}}_{\text{e}{\text{g}}^{\prime }}\times \text{O}{\text{R}}_{{\text{e}}^{\prime }\text{g}}, \end{array}$$

where OR_eg_ is the odds ratio for the presence of both cagA-positive status and risk genotypes of XRCC7 rs#7003908 (OR > 1), OR_eg′_ is the odds ratio for cagA-positive status for patients with the low-risk XRCC7 genotype, and OR_e′g_ is the odds ratio for the high risk XRCC7 genotype in patients with cagA-negative status. A *P* value of <0.05 was considered statistically significant in this study.

## 3. Results

### 3.1. Demographic Characteristics of the Subjects

Our final analysis included 642 GAA cases and 927 controls (Table 1). The characteristics of cases and controls showed that there were no significant differences between cases and controls in terms of the distribution of age, sex, race, and smoking and drinking status as a result of individual matching. These results suggest the GAA patient data were comparable to the control data.

### 3.2. CagA Status and GAA Risk

Table 2 summarizes information on cagA status for the entire study population. The frequency of cagA-positive status among cases (66.5%) was higher than among controls (22.3%), with *P*  value < 0.01. Conditional logistic regression analysis exhibited that, compared to individuals with cagA-negative status, these with cagA-positive status would feature higher GAA risk (OR = 6.38).

### 3.3. XRCC7 rs#7003908 Polymorphism and GAA Risk

The genotypic distribution of XRCC7 rs#7003908 for both cases and controls is shown in Table 3. The genotypic distribution of this gene in controls was in Hardy-Weinberg equilibrium. The frequencies of rs#7003908 G allele were higher in cases (0.53) than controls (0.28). 

The adjusted OR for GAA among those heterozygous for XRCC7 rs#7003908 T and G allele (XRCC7-TG) versus those homozygous for XRCC7 rs#7003908 T alleles (XRCC7-TT) was 2.80 (95% CI, 2.15–3.64). The corresponding OR for those homozygous for XRCC7 rs#7003908 G alleles (XRCC7-GG) was 5.13 (95% CI, 3.71–7.11). Thus, GAA risk was associated with the number of XRCC7 rs#7003908 G alleles.

To assess possible interactive effects of matching factors and XRCC7 rs#7003908 polymorphism on GAA risk, we analyzed this polymorphism stratified by matching factors such as age, sex, ethnicity, alcohol stratus, and smoking status. The results demonstrated similar risk estimates of around 3-fold increased GAA risk for the genotypes with XRCC7 rs#7003908 G alleles (data not shown). Likelihood ratio tests for these matching factors did not substantially modulate the effect of this polymorphism on cancer risk.

### 3.4. Joint Effects of CagA Status and XRCC7 Polymorphism on GAA Risk

The joint effects of cagA status and XRCC7 genotypes on GAA risk are provided in Table 4. In this analysis, we used as a reference the lowest risk group: those who had XRCC7-TT and cagA-negative status. Results showed that cagA-positive status increased GAA risk; moreover, this risk was more pronounced among subjects with XRCC7 risk genotypes. There was evidence of multiplicatively interactive effects of genotypes and cagA status on GAA risk (20.85 > (3.95 × 2.40)) according to the previously published formula (OR_eg_ > (OR_eg′_ × OR_e′g_)) [20–22].

### 3.5. Joint Effects of XRCC7 Polymorphism and XRCC3 Polymorphism on GAA Risk

Because our previous study showed that XRCC3 rs#861539 polymorphism [18], another DNA repair gene involved in DSBR, modifies Guangxiese GAA risk, we explored possible gene-gene interaction between XRCC7 and XRCC3. Because of the small number of subjects with both the homozygotes of the XRCC3 rs#861539 T alleles (XRCC3-TT) and XRCC7-GG, the combination of these two genes was divided into four strata (Table 5). Results exhibited that individuals with both XRCC7-TG/GG and genotypes of the XRCC3 rs#861539 T alleles (XRCC3-CT/TT), in comparison with those with both XRCC7-TT and homozygotes of the XRCC3 rs#861539 T alleles (XRCC3-TT), might face a higher GAA risk (OR, 3.32, 95% CI, 2.54–4.34). A likelihood ratio test showed that there were multiplicatively interactive effects of XRCC7 and XRCC3 on the GAA risk (*P*_interaction_ = 1.12 × 10^−37^).

### 3.6. XRCC7 Polymorphism and Lauren's Classification

Because Lauren's classification of gastric cancer is related to the prognosis and the differentiation of this malignant tumor [23], we analyzed the distribution of XRCC7 rs#7003908 genotypes in the different Lauren's type: intestinal type and diffuse type (Table 6). We observed a higher frequency of genotypes with mutant alleles (XRCC7-TG/GG) among GAA patients with diffuse type cancer (93.5%) than among those with intestinal type cancer (70.0%). Logistic regression analysis showed that the corresponding risk value was 5.89 (2.89–1.98), with *P* value of 1.01 × 10^−6^. 

Additionally, we also investigated the relationship between this polymorphism and primary tumor sites (cardia versus noncardia) among 120 patients with cardia gastric cancer and 642 GAA patients. Among these cases with cardia gastric cancer, the frequency of XRCC7-TT, -TG, and -GG genotype was 52.3% (63/120), 35.8% (43/120), and 11.7% (14/120), respectively. This suggested that GAA patients, compared with those with cardiac gastric cancer, had higher mutant frequency of XRCC7 rs#7003908. Risk analysis next showed the corresponding ORs (95% CIs) were 2.63 (1.70–4.05) for XRCC7-TG and 5.48 (2.96–10.15) for XRCC7-GG, respectively. However, XRCC7 rs#7003908 polymorphism did not modify cardia gastric cancer risk (*P* > 0.05, data not shown).

## 4. Discussion

To the best of our knowledge, there has been no report on the association between XRCC7P and the risk of GAA, especially from high *H. pylori* infection areas. In this hospital-based case-control study, we analyzed the association between aforementioned polymorphism and the risk of GAA among Guangxi population from a high* H. pylori* infection area. Overall, we found that the genotypes with XRCC7 rs#7003908 G alleles had a substantial association with the increasing risk of GAA among Guangxi population (OR = 3.41). We also observed a significant interactive effect between XRCC7P and *H. pylori *infection status on GAA risk. These results might imply that this polymorphism may have functional significance in the carcinogenesis of *H. pylori* infection-related GAA.

GAA is one of the major cancer types in the Guangxi Zhuang Autonomous Region; the most environmental cause of which is high *H. pylori* infection among Guangxi population [3, 5]. Our previous studies [18, 19] and prevent report also show that GAA patients have higher rate of *H. pylori* infection. However, increasing epidemiological evidence has shown that although many people are infected with *H. pylori*, only a relatively small proportion of individuals with chronic* H. pylori *infection develop GAA [24]. This suggests possible individual susceptibility related to genetic factors such as DNA repair capacity [18, 19, 25, 26].

Among these known genetic factors, the NHEJ genes have been reported as a cancer-correlated genetic factor that plays an important role in the repair of DSBs resulting from exogenous attacks such as cagA [10, 27, 28]. XRCC7 (also known as PRKDC/HYRC/HYRC1) is an important DNA repair gene involved in the NHEJ pathway [13, 14, 29]. This gene is located on chromosome 8q11 and consists of 85 exons and 86 introns [29]. The encoded protein of XRCC7 consists of more than 4,000 amino acid residues and constitutes the large catalytic subunit of DNA-PK complex (DNA-PKcs). When DNA-PKcs is recruited to the site of DSBs by the Ku70/Ku80 heterodimer (another subunit of DNA-PK complex), DNA-PK complex then changes into its active form and subsequently initiates NHEJ repair [11, 14]. Murine mutants defective in the XRCC7 have nondetectable DNA-PK activity, suggesting XRCC7 is required for NHEJ pathway [30].

More than one hundred polymorphisms have been reported in the XRCC7 gene (dbSNP in NCBI database), some of which are correlated with malignant tumors such as liver cancer [16, 17, 31]. In the present study, we only analyzed XRCC7P, primarily because several studies have shown that this polymorphism may influence the expression of XRCC7 gene and consequently increase the risk of cancers, such as liver, bladder, and prostate cancer [15, 17, 32, 33]. For example, Long et al. investigated the risk effects of XRCC7P on hepatocellular carcinoma (HCC) and found that these individuals carrying XRCC7-GG would face higher cancer risk than those with XRCC7-TT (adjusted OR = 5.04, *P* < 0.001) [17]. Similarly, Mandal et al. and Wang et al. also found that this polymorphism might modify the risk of prostate cancer and bladder cancer [32, 33]. Not only did our current data support this hypothesis in view of XRCC7P modifying tumor risk, but we also found that this polymorphism would be able to interact with environmental carcinogens in the process of GAA induced by *H. pylori* infection. A recent report has shown that XRCC7P increases the levels of DNA adducts and decreases the capacity of NHEJ pathway [17]. Those results suggest that this polymorphism may modulate the normal XRCC7 function, and consequently low NHEJ capacity resulting from XRCC7 function might progress gastric carcinogenesis [34]. Thus, the DNA damage induced by cagA cannot be repaired effectively and duly and, subsequently, may induce genic mutation and gastric mucosal cells canceration. Therefore, XRCC7P may play a role in the carcinogenesis of Guangxiese GAA. In addition, we found some evidence of XRCC7-XRCC3 interactive effects on GAA risk, possibly because this gene-gene interaction results in a more obvious decrease in the NHEJ capacity and consequently correlates with a higher risk for GAA.

In this study, we also investigated the association between XRCC7P and the pathological features of gastric cancer. We observed that these individuals having the mutant genotypes of XRCC7P would feature higher risk of diffuse-type gastric cancer than those with wild genotype of XRCC7P, suggesting that this polymorphism might be correlated with the dedifferentiation of gastric cancer. Furthermore, we also found some evidence of XRCC7P correlating with primary tumor sites of gastric cancer. This is possibly because of the following the reasons: (a) DNA damage induced by *H. pylori* infection emerging in noncardia gastric mucosa, especially gastric antrum mucosa, because *H. pylori* mainly infects noncardia gastric mucosa [35]; (b) high *H. pylori* infection rate among Guangxi population [19]; and (c) tissue tumorigenesis and tumor progression easily occurring in these tissues or cells with lots of DNA damage, especially under the condition of low DNA repair capacity [36–38].

When investigating the association between genetic polymorphisms and *environmental factors*-related cancer risk, it is important to collect sufficiently large samples to test for gene-environment interaction and to avoid the effects of confounders [21]. In this study, the effects of possible confounder, including age, sex, race, and smoking and drinking status, were controlled with an individually matched design. In the stratified analysis, no interactive effects were found, suggesting that these factors do not modify the correlation between XRCC7P and GAA related to *H. pylori* infection.

In conclusion, this study is, to the best of our knowledge, the first report that describes the polymorphisms in the NHEJ gene XRCC7 and its correlations with *H. pylori* infection-related GAA risk. We find some evidence to imply that the genotypes of XRCC7 with rs#7003908 G alleles may increase the risk of *H. pylori* infection-related GAA and the DSBR pathway may play an important role in the mechanism of action of this genotoxin. However, there were several limitations to our study. Although our findings were based on relatively large numbers, potential selection bias might have occurred because the selection of control subjects in our study was hospital-based. Despite XRCC7P being analyzed, we did not investigate the genetic polymorphisms of other DNA repair genes involved in the DSBR such as XRCC5 [11]. Additionally, the relatively small sample size may not have enough power to analyze gene-gene and/or gene-environment interaction. Thus, more genes deserve further elucidation based on a population sample and the combination of genes and *H. pylori *infection information.

## Figures and Tables

**Table 1:** Demographic details of GAA cases and controls.

	Controls (*n* = 927)		Cases (*n* = 642)		*χ* ^2^	*P*
	*n*	%	*n*	%		
Gender					0.17	0.68
Man	664	71.6	466	72.6		
Female	263	28.4	176	27.4		
Age (years)^a^					7.61	0.47
≤35	59	6.4	49	7.6		
36–40	96	10.4	57	8.9		
41–45	112	12.1	58	9.0		
46–50	119	12.8	91	14.2		
51–55	130	14.0	106	16.5		
56–60	153	16.5	107	16.7		
61–65	90	9.7	59	9.2		
66–70	113	12.2	73	11.4		
≥71	55	5.9	42	6.5		
Race					0.90	0.34
Han	522	56.3	346	53.9		
Zhuang	405	43.7	296	46.1		
Smoking status					0.07	0.79
No	290	31.3	205	31.9		
Yes	637	68.7	437	68.1		
Drinking status					0.58	0.45
No	352	38.0	256	39.9		
Yes	575	62.0	386	60.1		

^a^The mean ± S.D. ages were 53.30 ± 11.38 and 53.54 ± 12.02 for cases and controls, respectively.

**Table 2:** CagA status and the risk of GAA.

CagA status	Controls (*n* = 927)		Cases (*n* = 642)		OR (95% CI)^a^	*P*_trend_
	*n*	%	*n*	%		
Negative	720	77.7	215	33.5	Reference	
Positive	207	22.3	427	66.5	6.38 (5.03–8.09)	1.23 × 10^−52^

^a^OR conditional on matched set.

**Table 3:** XRCC7 rs#7003908 polymorphism and GAA risk.

rs#7003908	Controls (*n* = 927)		Cases (*n* = 642)		OR (95% CI)	*P*_trend_
	*n*	%	*n*	%		
Genotype						
TT	523	56.4	160	24.9	Reference	
TG	297	32.0	287	44.7	2.80 (2.15–3.64)^a^	2.14 × 10^−14^
GG	107	11.5	195	30.4	5.13 (3.71–7.11)^a^	7.34 × 10^−23^
TG/GG	404	43.6	482	75.1	3.41 (2.68–4.35)^a^	4.09 × 10^−23^
Allele						
T	1343	72.4	607	47.3	Reference	
G	511	27.6	677	52.7	2.93 (2.52–3.40)	4.41 × 10^−45^

^a^OR conditional on matched set adjusted for cagA status.

**Table 4:** Joint effects of cag A status and XRCC7 rs#7003908 polymorphism on GAA risk.

cag A	rs#7003908	Controls (*n* = 927)		Cases (*n* = 642)		OR (95% CI)^a^	*P*_trend_
		*n*	%	*n*	%		
Negative	Genotype						
	TT	408	44.0	78	12.1	Reference	
	TG	226	24.4	87	13.6	2.10 (1.48–2.97)	3.21 × 10^−5^
	GG	86	9.3	50	7.8	3.24 (2.11–4.98)	7.86 × 10^−8^
	TG/GG	312	33.7	137	21.3	2.40 (1.75–3.30)	6.25 × 10^−8^

Positive	TT	115	12.4	82	12.8	3.95 (2.71–5.96)	9.24 × 10^−13^
	TG	71	7.7	200	31.2	15.74 (10.89–22.77)	1.50 × 10^−48^
	GG	21	2.3	145	22.6	38.49 (22.82–64.93)	1.31 × 10^−42^
	TG/GG	92	9.9	345	53.7	20.85 (14.84–29.30)	1.14 × 10^−68^

^a^OR conditional on matched set.

**Table 5:** Joint effects of XRCC7 rs#7003908 polymorphism and XRCC3 rs#861539 on GAA risk.

rs#861539	rs#7003908	Controls (*n* = 927)		Cases (*n* = 642)		OR (95% CI)^a^	*P*_trend_
		*n*	%	*n*	%		
CC	TT	477	51.5	136	21.2	Reference	
	TG/GG	113	12.2	94	14.6	2.73 (1.89–3.94)	9.39 × 10^−8^

CT/TT	TT	46	5.0	24	3.7	0.60 (0.34–1.05)	0.08
	TG/GG	291	31.4	388	60.4	3.32 (2.54–4.34)	2.15 × 10^−18^

^a^OR conditional on matched set adjusted for cagA status.

Likelihood ratio test for gene-gene interaction, OR = 1.49 (1.39–1.56), *P* = 1.12 × 10^−37^.

**Table 6:** XRCC7 rs#7003908 polymorphism and Lauren's classification.

rs#7003908	Intestinal type (*n* = 504)		Diffuse type (*n* = 138)		OR (95% CI)^a^	*P*_trend_
	*n*	%	*n*	%		
TT	151	30.0	9	6.5	Reference	
TG	228	45.2	59	42.8	4.18 (2.00–8.75)	1.49 × 10^−4^
GG	125	24.8	70	50.7	9.12 (4.34–19.19)	5.69 × 10^−9^
TG/GG	353	70.0	129	93.5	5.89 (2.89–1.98)	1.01 × 10^−6^

^a^Adjusted by age, sex, race, smoking and drinking status, and cagA status.

## Abbreviations

GAA: Gastric antrum adenocarcinoma
*H. pylori*: *Helicobacter pylori*
DSBR: Double strand break repair
OR: Odds ratio
PCR: Polymerase chain reaction
XRCC7: X-ray repair cross-complementing group 7
XRCC3-CC: The homozygotes of XRCC3 rs#861539 C alleles
XRCC3-CT: The heterozygotes of XRCC3 rs#861539 C and T allele
XRCC3-CT/TT: The genotypes with XRCC3 rs#861539 T alleles
XRCC3-TT: The homozygote of XRCC3 rs#861539 T alleles
XRCC7-TT: The homozygotes of XRCC7 rs#7003908 T alleles
XRCC7-TG: The heterozygotes of XRCC7 rs#7003908 T and G allele
XRCC7-GG: The homozygote of XRCC7 rs#7003908 G alleles
XRCC7-TG/GG: The genotypes with XRCC7 rs#7003908 G alleles.
